# Developing a Time-Adaptive Prediction Model for Out-of-Hospital Cardiac Arrest: Nationwide Cohort Study in Korea

**DOI:** 10.2196/28361

**Published:** 2021-07-05

**Authors:** Ji Woong Kim, Juhyung Ha, Taerim Kim, Hee Yoon, Sung Yeon Hwang, Ik Joon Jo, Tae Gun Shin, Min Seob Sim, Kyunga Kim, Won Chul Cha

**Affiliations:** 1 Department of Digital Health Samsung Advanced Institute for Health Science & Technology Sungkyunkwan University Seoul Republic of Korea; 2 Department of Computer Science Indiana University Bloomington, IN United States; 3 Department of Emergency Medicine Samsung Medical Center Sungkyunkwan University School of Medicine Seoul Republic of Korea; 4 Statistics and Data Center Research Institute for Future Medicine Samsung Medical Center Seoul Republic of Korea; 5 Health Information and Strategy Center Samsung Medical Center Seoul Republic of Korea

**Keywords:** out-of-hospital cardiac arrest, Republic of Korea, machine learning, artificial intelligence, prognosis, cardiology, prediction model

## Abstract

**Background:**

Out-of-hospital cardiac arrest (OHCA) is a serious public health issue, and predicting the prognosis of OHCA patients can assist clinicians in making decisions about the treatment of patients, use of hospital resources, or termination of resuscitation.

**Objective:**

This study aimed to develop a time-adaptive conditional prediction model (TACOM) to predict clinical outcomes every minute.

**Methods:**

We performed a retrospective observational study using data from the Korea OHCA Registry in South Korea. In this study, we excluded patients with trauma, those who experienced return of spontaneous circulation before arriving in the emergency department (ED), and those who did not receive cardiopulmonary resuscitation (CPR) in the ED. We selected patients who received CPR in the ED. To develop the time-adaptive prediction model, we organized the training data set as ongoing CPR patients by the minute. A total of 49,669 patients were divided into 39,602 subjects for training and 10,067 subjects for validation. We compared random forest, LightGBM, and artificial neural networks as the prediction model methods. Model performance was quantified using the prediction probability of the model, area under the receiver operating characteristic curve (AUROC), and area under the precision recall curve.

**Results:**

Among the three algorithms, LightGBM showed the best performance. From 0 to 30 min, the AUROC of the TACOM for predicting good neurological outcomes ranged from 0.910 (95% CI 0.910-0.911) to 0.869 (95% CI 0.865-0.871), whereas that for survival to hospital discharge ranged from 0.800 (95% CI 0.797-0.800) to 0.734 (95% CI 0.736-0.740). The prediction probability of the TACOM showed similar flow with cohort data based on a comparison with the conventional model’s prediction probability.

**Conclusions:**

The TACOM predicted the clinical outcome of OHCA patients per minute. This model for predicting patient outcomes by the minute can assist clinicians in making rational decisions for OHCA patients.

## Introduction

Out-of-hospital cardiac arrest (OHCA) is a common but serious public health issue [1]. Globally, 55 in 100,000 people experience OHCA every year [2]. Despite vigorous and continuous efforts, the survival rate of OHCA patients is poor in many countries. Thus, OHCA is a critical medical problem with a poor prognosis [3,4].

Predicting the prognosis of OHCA patients can assist clinicians in deciding whether to provide treatment or use hospital resources [5,6]. Appropriate decisions allow better utilization of hospital resources, reduce medical expenses considerably, and increase the availability of care for other patients [7,8]. Furthermore, determining the termination of resuscitation for OHCA patients is an important issue because of the limited resources of hospitals. Prediction-based decision-making for using mechanical circulatory support devices or for executing early coronary angiography could improve the clinical outcomes of patients [9-11].

Some studies have proposed prediction models to predict clinical outcomes of OHCA patients using Utstein data [12,13]. These studies have shown that basic Utstein variables are associated with patient clinical outcomes. However, these studies determined patient outcomes after hospitalization and not in real time. Other studies predicting the prognosis of OHCA patients also predicted the long-term outcomes of patients, such as 1-year survival and posthospital outcomes, using various laboratory results [14,15]. Our study differs from previous studies in that the decision time and the prediction available time in our study are different.

In this study, we developed a machine learning–based time-adaptive conditional prediction model (TACOM) using the concept of a time-adaptive cohort to predict outcomes among OHCA patients every minute. We compared the random forest, LightGBM, and artificial neural network algorithms to develop a precise prediction model. The time-adaptive cohort was derived from the concept of censoring. We used the TACOM, based on the concept of the time-adaptive cohort, to compare the prediction probability with the conventional model to demonstrate the possibility of predicting patients’ clinical outcomes every minute during cardiopulmonary resuscitation (CPR). To the best of our knowledge, this study is the first to predict the prognosis of OHCA patients by the minute.

## Methods

### Data Sets

We performed a nationwide retrospective observational cohort study using data from the Korea OHCA Registry (KOHCAR). The KOHCAR was constructed by the Korea Centers for Disease Control and Prevention (CDC) in collaboration with the Central Fire Services (CFS). We integrated the emergency medical services (EMS) run sheet, EMS CPR registry, and dispatch CPR registry into the EMS-assessed cardiac arrest database of the CFS [16,17]. Data collection was based on the Utstein style and Resuscitation Outcome Consortium Project customized for local conditions. To assess the quality of data, we held monthly meetings with field investigators and the CDC data quality control team [18]. Trained managers visited the hospitals to review the medical records and complete the database. Additionally, they contacted the patients to verify information about the outcomes [19-21]. The Korea CDC approved the use of all data.

In this study, the KOHCAR data set from January 1, 2013, to December 31, 2017, was used for the training set, and the data set from January 1, 2018, to December 31, 2018, was used for the test set. Patients who experienced return of spontaneous circulation (ROSC) before arriving in the emergency department (ED), those who did not receive CPR in the ED, and those with missing information were excluded. The institutional review board of the Samsung Medical Center approved this study.

### Predictor Variables and Endpoints

Predictor variables included patient demographics, occurrence-related information, and hospital treatment information available at the time of the patient’s arrival in the ED. The demographics included age and sex. Occurrence-related information included place (public or private), OHCA etiology, witness of the event, bystander CPR, prehospital CPR, patient’s act at the time of OHCA, prehospital electrocardiography (ECG) rhythm, prehospital defibrillation, and history of hypertension, diabetes, heart disease, renal disease, respiratory disease, stroke, and dyslipidemia. Hospital treatment information included EMS-to-ED time, initial ECG rhythm at the ED, defibrillation, and the place of the first defibrillation.

The outcomes of this model were patient survival to hospital discharge and a good neurological outcome. For patient survival to hospital discharge, we considered patients whose ED treatment resulted in being discharged or whose hospitalization resulted in being discharged, voluntarily discharged, or transferred. A good neurological outcome was defined as a cerebral performance category of 1 or 2.

### Data Processing

We used both numerical and categorical variables. The patient age and EMS-to-ED time were numerical variables. A scaling method was used for numerical variables to increase the ability of the prediction model. For the age values, we used a standard scaler that could normalize each feature by removing its mean and scaling its variance to 1. For EMS-to-ED time values, we used a robust scaler that utilized quantile information to scale each feature through the application of an inverse cumulative distribution function. For categorical variables, we used one-hot encoding to remove integer-encoded variables and then added new binary variables for each unique integer value.

### Model Development

To develop a real-time outcome prediction model, we included the per minute data of patients with ongoing CPR. When predicting the clinical outcome of patients in real time, we deemed it unreasonable to predict the outcome of patients whose conditions had been determined. In other words, it is reasonable to predict the outcome of patients whose condition has not been previously determined [16]. We trained the TACOM with every single data set by minute. Data set D(t) was defined as follows:

*D* (*T*_2_|*T*_1_>*t*) **(1)**

where *t* is a minute from 0 to 60, *T*_1_>t indicates patients whose CPR duration is longer than time *t*, and *T*_2_ indicates patients who had a clinical outcome (survival to hospital discharge or good neurological recovery). The TACOM system included 61 models trained with different data sets over time. CPR duration was not used as a machine learning feature in the model. The model’s training data set cohort varied for each minute according to the duration of CPR.

Additionally, we developed three models, namely, random forest, LightGBM, and artificial neural networks, to select the best performance model. Both the deep learning algorithm and machine learning algorithm performed well in the health care domain [22,23]. We compared the area under the receiver operating characteristic curve (AUROC) for model performance and chose LightGBM as our final model. We have provided the AUROC of the other models in Multimedia Appendix 1. LightGBM, an open-source algorithm by Microsoft, is an advanced model of the ensemble algorithm for speeding up the training process and reducing memory consumption. In general, the ensemble model has shown remarkable performance for the classification of structured data. It generates several classifiers and combines predictions to derive a final prediction. Ensembles have the following two main types: bagging and boosting. In the bagging algorithm, each training set is constructed by forming a bootstrap replicate of the original training set. In the boosting algorithm, the model maintains a set of weights over the original training set and adjusts these weights after each classifier is learned by the base algorithm [24].

We utilized a widely used parameter optimization algorithm, the grid search, to determine the best combination of the three hyperparameters in LightGBM. Referring to the technical documents provided by Microsoft, first, we found the value of “max_depth,” which specifies the depth limit of the tree. Through grid search, we selected the value that had the highest AUROC among the values from 1 to 10. After that, we found the value of “num_leaves” that controls the complexity of the tree model. Theoretically, since the number of leaves should be smaller than 2^“max_depth”^, one of the values from 100 to 900 was selected through the grid search. Finally, we found “min_data_in_leaf,” an essential parameter to prevent over-fitting. Its value ranges from 100 to 1000; we found an appropriate value through the grid search. The AUROC of each value is shown in Multimedia Appendix 2.

### Statistical Analysis

For the descriptive statistics, means and SDs were used for continuous variables, and frequencies and percentages were used for categorical variables. The *t* test and chi-squared test were performed to determine the mean differences between the derivation and test sets. All tests were two-tailed with the statistical significance level set at *P*<.05. Additionally, the standardized mean difference (SMD) was used to measure the effect size of the two groups.

We used various metrics, including prediction probability, AUROC, and area under the precision-recall curve (AUPRC), to measure the metric of our prediction model, TACOM. Prediction probability was used to determine which model reflected reality. AUROC and AUPRC scores were used to measure the performance of the binary outcomes. We evaluated 95% CIs using bootstrapping with 1000 sampling iterations with replacement.

### Implementation

**Figure 1:**
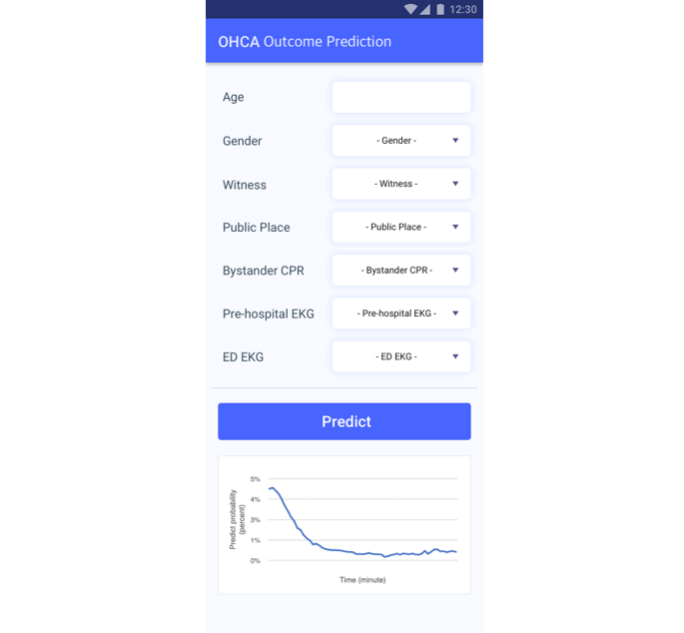
Simple user interface of the out-of-hospital cardiac arrest outcome prediction model.

Furthermore, we developed a simple user interface (Figure 1) for showing prediction probability using Android. By simply entering the input values, a patient’s outcomes can be predicted and visualized as a graph. We designed an application prototype. This application could provide information to the medical staff. The implemented software for model development included the Python programming language (version 3.8.5), Tensorflow framework (version 2.3.1), and scikit-learn (version 0.23.2). Using the Tensorflow framework, the predictive model could be extended to a mobile or web application.

### Code Availability

We published our prediction model on GitHub [25]. The codes that support the findings of this study are available on GitHub.

## Results

### Patient Selection

**Figure 2:**
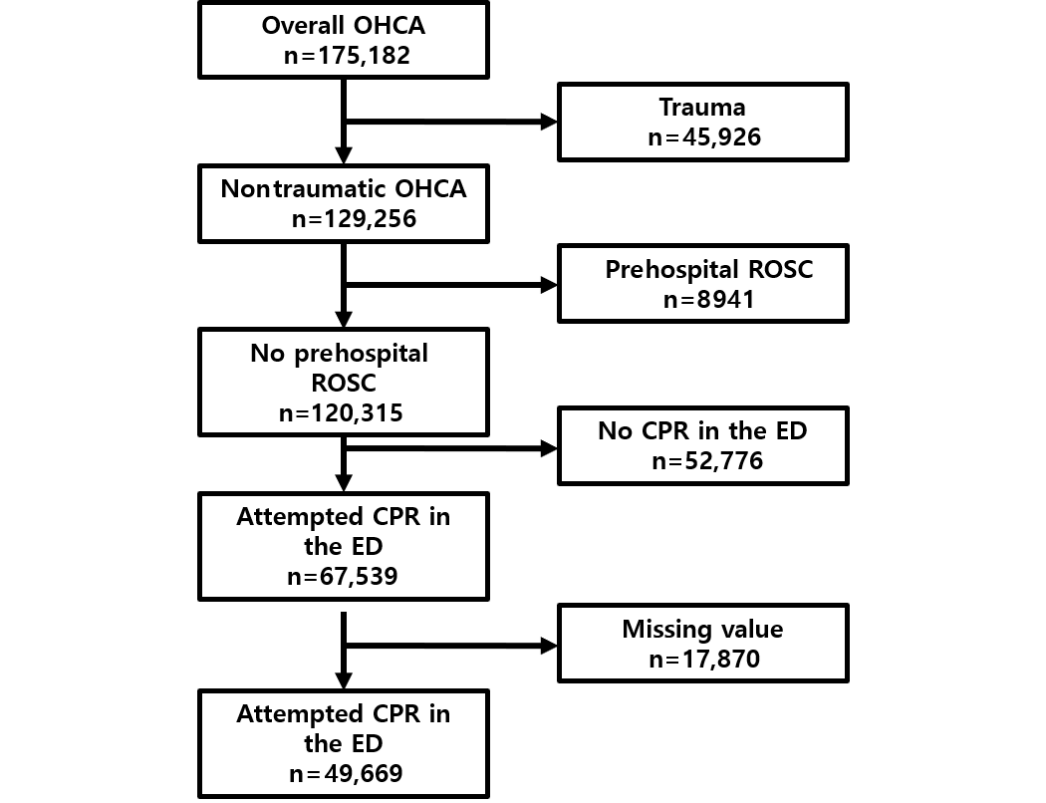
Subject selection process of OHCA patients. CPR: cardiopulmonary resuscitation; ED: emergency department; OHCA: out-of-hospital cardiac arrest; ROSC: return of spontaneous circulation.

Patients’ records from the KOHCAR were used as derivation and validation data sets. The KOHCAR held records for 175,182 patients from 2013 to 2018. After excluding trauma patients, patients who experienced ROSC in the prehospital stage, patients who did not receive CPR in the ED, and patients with missing values, we included 49,669 records in our study. We split the data set into 39,602 records from 2013 to 2017 to be used as the derivation set, and 10,067 records from 2018 to be used as the validation set. Figure 2 presents the diagram of patient selection from the KOHCAR.

### Patient Characteristics

Table 1 shows the baseline characteristics of the derivation and validation sets from the KOHCAR. Overall, the patients’ basic characteristics showed that the majority were men (32,049/49,669, 64.5%), and the average age was 67.0 years, with a quartile range of 57.0 to 79.0 years. The shockable rhythm rates in the ED and EMS were 4.4% (2170/49,669) and 10.2% (5,067/49,669), respectively.

**Table 1:** Basic characteristics of the study participants.

Variable		All (n=49,669)	Derivation data (n=39,602)	Validation data (n=10,067)	*P* value	SMD^a^
Age, mean (SD)		67.0 (18.8)	66.5 (19.3)	68.8 (16.6)	<.001	0.125
Female sex, n (%)		17,620 (35.5%)	13,932 (35.2%)	3688 (36.6%)	.007	0.030
Public place, n (%)		8030 (16.2%)	6517 (16.5%)	1513 (15.0%)	<.001	0.130
Witnessed, n (%)		30,314 (61.0%)	24,126 (60.9%)	3552 (35.3%)	<.001	0.190
Bystander CPR^b^, n (%)		9966 (20.1%)	7417 (18.7%)	2549 (25.3%)	<.001	0.333
**Cause, n (%)**					<.001	0.114
	Cardiogenic disease	45,792 (92.2%)	36,495 (92.2%)	9297 (92.4%)		
	Respiratory disease	381 (0.8%)	303 (0.8%)	78 (0.8%)		
	Nontraumatic bleeding	775 (1.6%)	558 (1.4%)	217 (2.2%)		
	Terminal cancer	459 (0.9%)	430 (1.1%)	29 (0.3%)		
	Sudden infant death syndrome	197 (0.4%)	147 (0.4%)	50 (0.5%)		
	Others	2065 (4.2%)	1,669 (4.2%)	396 (3.9%)		
**Initial ECG^c^ rhythm of EMS^d^, n (%)**					<.001	N/A^e^
	VF^f^	4732 (9.5%)	3603 (9.1%)	1129 (11.2%)		
	Pulseless VT^g^	335 (0.7%)	258 (0.7%)	77 (0.8%)		
	Asystole	15,820 (31.9%)	11,593 (29.3%)	4227 (42.0%)		
	PEA^h^	5406 (10.9%)	3637 (9.2)	1769 (17.6%)		
	Others	23,376 (47.0%)	20,511 (51.7%)	2865 (28.4%)		
**Initial ECG rhythm of ED^i^, n (%)**					<.001	N/A
	VF	1913 (3.9%)	1576 (4.0%)	337 (3.3%)		
	Pulseless VT	257 (0.5%)	218 (0.6%)	39 (0.4%)		
	Asystole	29,433 (59.3%)	23,532 (59.4%)	5901 (58.6%)		
	PEA	5615 (11.3%)	4031 (10.2%)	1584 (15.7%)		
	Others	12,451 (25.0%)	10,245 (25.8%)	2206 (22.0%)		
Anamnesis hypertension, n (%)		17,709 (35.7%)	13,886 (35.1%)	3823 (38.0%)	<.001	0.184
Anamnesis diabetes, n (%)		11,787 (23.7%)	9188 (23.2%)	2599 (25.8%)	<.001	0.209
Anamnesis heart disease, n (%)		8720 (17.6%)	6774 (17.1%)	1946 (19.3%)	<.001	0.059
Anamnesis renal disease, n (%)		3192 (6.4%)	2489 (6.3%)	703 (7.0%)	.02	0.031
Anamnesis respiratory disease, n (%)		3373 (6.8%)	2613 (6.6%)	760 (7.5%)	.003	0.037
Anamnesis stroke, n (%)		4261 (8.6%)	3333 (8.4%)	928 (9.2%)	.01	0.033
Anamnesis dyslipidemia, n (%)		1201 (2.4%)	863 (2.2%)	338 (3.4%)	<.001	0.073
EMS arrival (min), mean (SD)		45.9 (75.5)	44.7 (73.0)	50.6 (84.5)	<.001	0.074

^a^SMD: standardized mean difference.

^b^CPR: cardiopulmonary resuscitation.

^c^ECG: electrocardiography.

^d^EMS: emergency medical services.

^e^N/A: not applicable.

^f^VF: ventricular fibrillation.

^g^VT: ventricular tachycardia.

^h^PEA: pulseless electrical activity.

^i^ED: emergency department.

### Study Cohort

**Figure 3:**
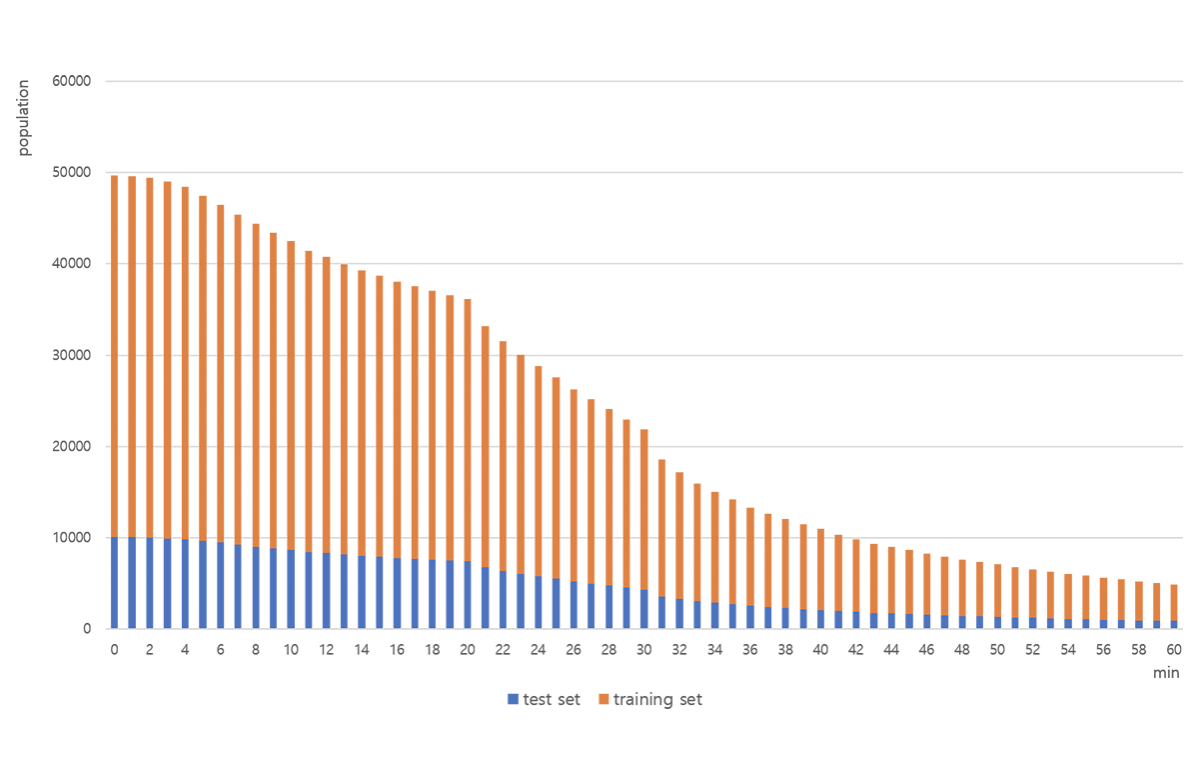
Population of every data set included in each minute from 0 to 60.

As shown in Figure 3, we divided our data set by CPR duration from 0 to 60 minutes. As time progressed, the models were trained using patients who were receiving CPR. In other words, patients who did not receive CPR were excluded. We did not use CPR duration as a feature but as a criterion that divided the data by minutes to apply the concept of time. The first model included 49,669 patients. The 30-minute model was trained using the data of 21,841 patients, indicating that the status of 27,828 patients in the ED had already been determined after 30 minutes.

### Model Performance

**Figure 4:**
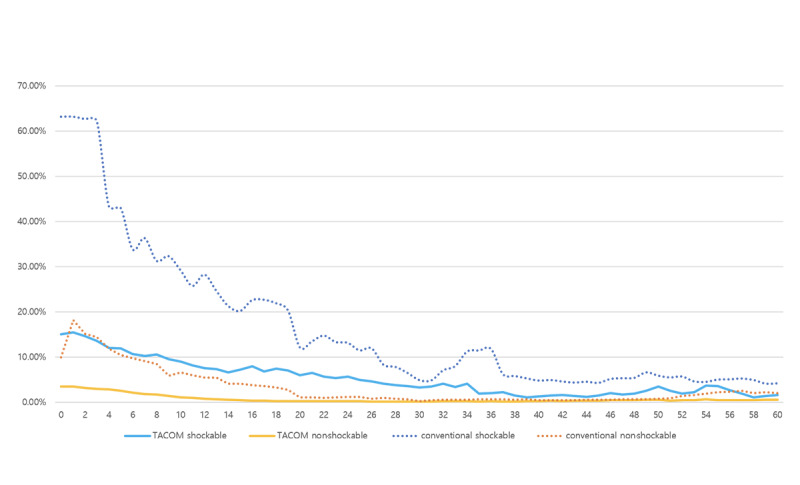
Prediction probability of the TACOM and conventional model for out-of-hospital cardiac arrest patients’ survival to hospital discharge. TACOM: time-adaptive conditional prediction model.

Figure 4 shows the prediction probability of the outcome (survival to hospital discharge) for the TACOM and that for a prediction model that uses CPR duration as a machine learning feature. Considering that the survival to hospital discharge rate of OHCA patients is about 5%, the TACOM reflected reality, whereas the prediction model that used CPR duration as a training feature was overly optimistic.

**Figure 5:**
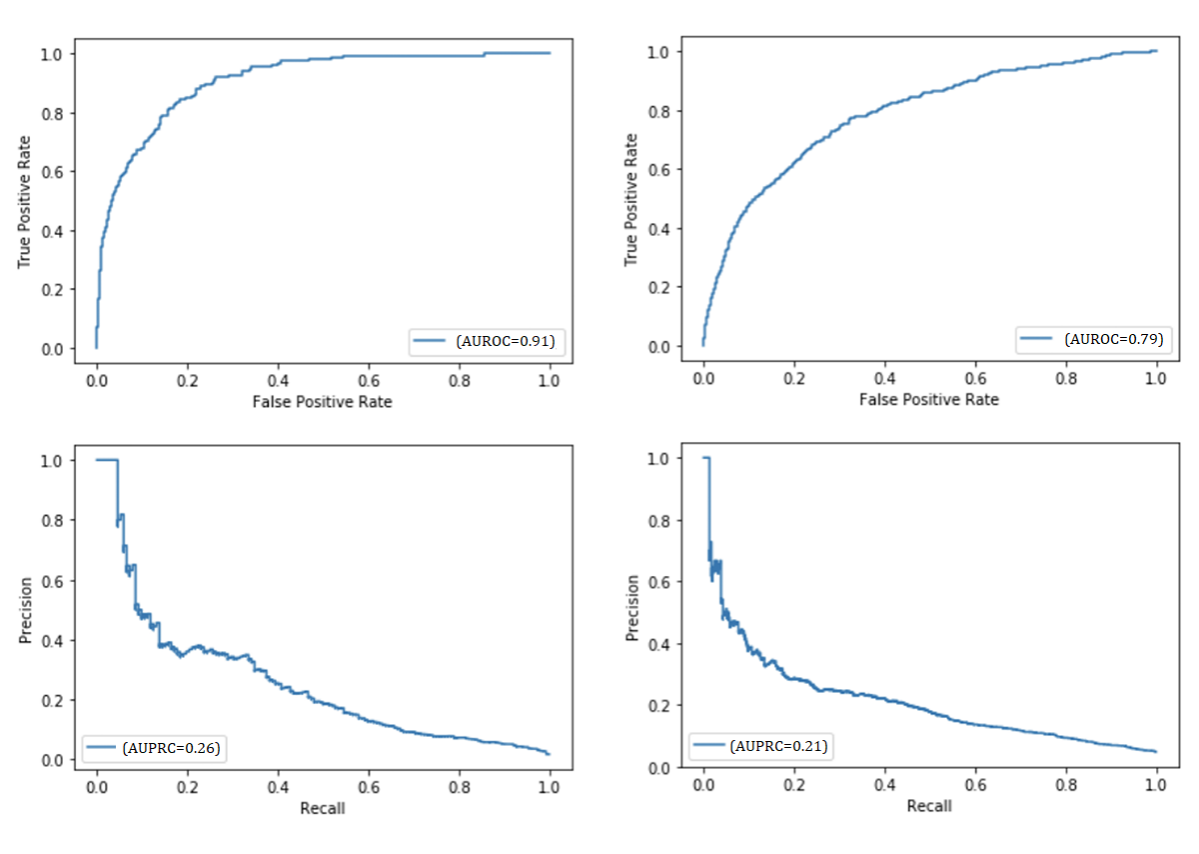
Area under the receiver operating characteristic curve (AUROC) and area under the precision-recall curve (AUPRC) of the time-adaptive conditional model for out-of-hospital cardiac arrest (OHCA) patients’ survival to hospital discharge at 2 minutes (right) and OHCA patients’ good neurological outcome at 2 minutes (left).

Figure 5 shows the receiver operating characteristic and precision-recall curves for the test set at 2 minutes, trained with data on patients whose outcomes had not been determined before 2 minutes. The AUROC and AUPRC of all the minutes from 0 to 30 are shown in Multimedia Appendix 3.

## Discussion

### Principal Findings

We developed a time-adaptive conditional model for predicting the clinical outcomes of OHCA patients using machine learning data from a large nationwide cohort registry. Our study demonstrated the possibility of real-time prediction among OHCA patients using a time-adaptive conditional model. The most important finding of this study was that the time-adaptive conditional model, in which CPR duration was not used as a feature but as a criterion to define the time-adaptive cohort, reflected real-world outcomes. The training data were divided by time according to the duration of CPR and trained separately for each time. Thus, the model was verified to be suitable for the real-time prediction of clinical outcomes.

The TACOM predicted the probability of survival of specific patients, updated every minute in a personalized manner. Hence, it could have practical application in the field. Currently, clinical decision-making is based on the personal experience of the medical staff or institutional guidelines; thus, decisions do not necessarily reflect the actual condition of each patient [17]. We used a machine learning–based model to calculate a patient’s clinical outcome in real time using the patient’s initial state. The clinical outcome prediction probability of the TACOM showed a similar flow to the actual clinical outcome rate of the cohort data.

We designed the TACOM to be different from the conventional model; the TACOM only used the initial information that could be obtained before CPR as a machine learning feature. Additionally, data, such as time arrival to EMS, time to EMS arrival at the ER, and lab results, were excluded. However, unlike with other models, the TACOM reflects the real environment using a time-adaptive cohort. We made the time-adaptive cohort from one big registry by censoring the data. Censoring is used when time-to-event information is not available such as in clinical trials or survival analysis in cancer treatment. We did not train all patients at once; however, we created the discriminative models by censoring the patients whose status was determined by the minute.

We aimed to apply and test the TACOM in the real world to make it practically useful and effective in the future. In this study, validation was performed using a subset of the data set. Prospective data collection and verification would be required and potentially achieved by developing an application that applies the TACOM. Furthermore, usability and utility evaluations for UX design are needed to identify whether the application is convenient and useful.

### Limitations

This study had some limitations. First, the cohort was organized in the Utstein style. The data were obtained from a nationwide cohort, and thus, some detailed characteristics reflecting the quality of CPR and patient responses were unavailable. Second, our model did not include long-term outcomes, such as 1-month or 1-year survival. Finally, given the absence of any significant difference between machine learning algorithms, various machine learning algorithms have not been included in this study.

### Conclusions

We developed a time-adaptive conditional model to predict the clinical outcomes of OHCA patients per minute. We found a suitable algorithm for the TACOM to predict the survival to hospital discharge and neurological recovery of OHCA patients at the minute level. This study showed the potential of the time-adaptive prediction model for resuscitation, which can be useful to medical staff for making appropriate and rational decisions.

## Appendix

Multimedia Appendix 1 Area under the receiver operating characteristic curve of the time-adaptive conditional model using the following three different methods: LightGBM, random forest, and deep learning.

Multimedia Appendix 2 Results of the grid search according to the three hyperparameters in LightGBM.

Multimedia Appendix 3 The area under the receiver operating characteristic curve and area under the precision-recall curve of the time-adaptive conditional model from 0 to 30 minutes.

## Abbreviations

AUPRC: area under the precision-recall curve
AUROC: area under the receiver operating characteristic curve
CDC: Centers for Disease Control and Prevention
CFS: Central Fire Services
CPR: cardiopulmonary resuscitation
ECG: electrocardiography
ED: emergency department
EMS: emergency medical services
KOHCAR: Korea Out-of-Hospital Cardiac Arrest Registry
OHCA: out-of-hospital cardiac arrest
ROSC: return of spontaneous circulation
TACOM: time-adaptive conditional prediction model
