# Supplementary material for: Developing a Time-Adaptive Prediction Model for Out-of-Hospital Cardiac Arrest: Nationwide Cohort Study in Korea
Source: J Med Internet Res. 2021 Jul 5;23(7):e28361. doi: 10.2196/28361 (PMC8406108; doi:10.2196/28361)
Supplement: Multimedia Appendix 1 [file jmir_v23i7e28361_app1.docx]

**Multimedia Appendix 1.** Area under the receiver operating characteristic curve of the time-adaptive conditional model using the following three different methods: LightGBM, random forest, and deep learning.

| Time (min) | LightGBM | Random Forest | Deep learning |
| --- | --- | --- | --- |
|  |  |  |  |
| 0 | 0.815 | 0.798 | 0.768 |
| 1 | 0.818 | 0.790 | 0.787 |
| 2 | 0.813 | 0.794 | 0.786 |
| 3 | 0.810 | 0.782 | 0.791 |
| 4 | 0.809 | 0.781 | 0.783 |
| 5 | 0.813 | 0.784 | 0.767 |
| 6 | 0.805 | 0.778 | 0.788 |
| 7 | 0.802 | 0.794 | 0.779 |
| 8 | 0.810 | 0.792 | 0.778 |
| 9 | 0.817 | 0.788 | 0.766 |
| 10 | 0.811 | 0.793 | 0.791 |
| 11 | 0.822 | 0.766 | 0.779 |
| 12 | 0.828 | 0.796 | 0.750 |
| 13 | 0.809 | 0.794 | 0.792 |
| 14 | 0.810 | 0.785 | 0.777 |
| 15 | 0.813 | 0.783 | 0.804 |
| 16 | 0.819 | 0.801 | 0.791 |
| 17 | 0.808 | 0.803 | 0.805 |
| 18 | 0.817 | 0.798 | 0.802 |
| 19 | 0.802 | 0.772 | 0.786 |
| 20 | 0.800 | 0.783 | 0.744 |
| 21 | 0.795 | 0.774 | 0.765 |
| 22 | 0.784 | 0.761 | 0.757 |
| 23 | 0.775 | 0.764 | 0.714 |
| 24 | 0.780 | 0.776 | 0.779 |
| 25 | 0.775 | 0.726 | 0.747 |
| 26 | 0.762 | 0.728 | 0.703 |
| 27 | 0.759 | 0.739 | 0.772 |
| 28 | 0.757 | 0.703 | 0.742 |
| 29 | 0.777 | 0.749 | 0.720 |
| 30 | 0.765 | 0.751 | 0.683 |
