# Supplementary material for: Developing a Time-Adaptive Prediction Model for Out-of-Hospital Cardiac Arrest: Nationwide Cohort Study in Korea
Source: J Med Internet Res. 2021 Jul 5;23(7):e28361. doi: 10.2196/28361 (PMC8406108; doi:10.2196/28361)
Supplement: Multimedia Appendix 2 [file jmir_v23i7e28361_app2.docx]

**Multimedia Appendix 2.** Results of the grid search according to the three hyperparameters in LightGBM.

| **max_depth** | **AUROC** |
| --- | --- |
| **10** | **0.76176545** |
| **20** | **0.75627331** |
| **30** | **0.75627331** |
| **40** | **0.75627331** |
| **50** | **0.75627331** |
| **60** | **0.75627331** |
| **70** | **0.75627331** |
| **80** | **0.75627331** |
| **90** | **0.75627331** |

Area under the receiver operating characteristic curve (AUROC) of the TACOM according to the value of max_depth

| **num_leaves** | **AUROC** |
| --- | --- |
| 100 | 0.73910103 |
| 200 | 0.73453965 |
| 300 | 0.73395443 |
| 400 | 0.74012161 |
| 500 | 0.74012161 |
| 600 | 0.74012161 |
| 700 | 0.74012161 |
| 800 | 0.74012161 |
| 900 | 0.74012161 |

Area under the receiver operating characteristic curve (AUROC) of the TACOM according to the value of num_leaves

| **min_data_in_leaf** | **AUROC** |
| --- | --- |
| 100 | 0.76019147 |
| 200 | 0.78039591 |
| 300 | 0.78306362 |
| 400 | 0.78728281 |
| 500 | 0.78502306 |
| 600 | 0.7872158 |
| 700 | 0.78932294 |
| 800 | 0.7892851 |
| 900 | 0.79222707 |

Area under the receiver operating characteristic curve (AUROC) of the TACOM according to the value of min_data_in_leaf
