# Supplementary material for: Developing a Time-Adaptive Prediction Model for Out-of-Hospital Cardiac Arrest: Nationwide Cohort Study in Korea
Source: J Med Internet Res. 2021 Jul 5;23(7):e28361. doi: 10.2196/28361 (PMC8406108; doi:10.2196/28361)
Supplement: Multimedia Appendix 3 [file jmir_v23i7e28361_app3.docx]

**Multimedia Appendix 3**. The area under the receiver operating characteristic curve and area under the precision-recall curve of the time-adaptive conditional model from 0 to 30 minutes.

| Good neurology outcome |  | Survival to hospital discharge |  |
| --- | --- | --- | --- |
| AUROC | AUPRC | AUROC | AUPRC |
| 0.91[0.91,0.910] | 0.237[0.24,0.244] | 0.797[0.797,0.798] | 0.238[0.238,0.241] |
| 0.91[0.91,0.910] | 0.248[0.25,0.254] | 0.797[0.797,0.798] | 0.236[0.237,0.240] |
| 0.909[0.909,0.91] | 0.257[0.258,0.263] | 0.787[0.786,0.788] | 0.213[0.214,0.216] |
| 0.912[0.912,0.914] | 0.237[0.24,0.244] | 0.791[0.790,0.791] | 0.210[0.209,0.211] |
| 0.909[0.909,0.91] | 0.192[0.196,0.201] | 0.788[0.787,0.789] | 0.210[0.211,0.213] |
| 0.91[0.91,0.910] | 0.216[0.221,0.225] | 0.790[0.790,0.791] | 0.200[0.201,0.204] |
| 0.91[0.909,0.910] | 0.196[0.198,0.203] | 0.780[0.779,0.780] | 0.188[0.188,0.190] |
| 0.917[0.915,0.917] | 0.183[0.188,0.193] | 0.784[0.782,0.784] | 0.184[0.185,0.188] |
| 0.920[0.920,0.922] | 0.188[0.191,0.196] | 0.789[0.788,0.790] | 0.174[0.175,0.178] |
| 0.922[0.920,0.922] | 0.188[0.196,0.201] | 0.795[0.795,0.797] | 0.179[0.179,0.181] |
| 0.922[0.92,0.922] | 0.193[0.2,0.206] | 0.778[0.777,0.779] | 0.172[0.173,0.176] |
| 0.910[0.91,0.912] | 0.171[0.182,0.187] | 0.793[0.792,0.793] | 0.168[0.168,0.171] |
| 0.91[0.91,0.912] | 0.192[0.203,0.209] | 0.805[0.804,0.806] | 0.152[0.154,0.157] |
| 0.909[0.907,0.91] | 0.178[0.188,0.195] | 0.798[0.797,0.799] | 0.136[0.136,0.139] |
| 0.9[0.899,0.902] | 0.19[0.196,0.203] | 0.787[0.786,0.789] | 0.124[0.127,0.130] |
| 0.894[0.893,0.895] | 0.181[0.188,0.195] | 0.792[0.791,0.793] | 0.116[0.118,0.121] |
| 0.889[0.887,0.89] | 0.188[0.188,0.196] | 0.807[0.806,0.809] | 0.122[0.125,0.129] |
| 0.899[0.895,0.899] | 0.182[0.187,0.193] | 0.793[0.792,0.794] | 0.117[0.120,0.123] |
| 0.902[0.900,0.904] | 0.18[0.187,0.195] | 0.783[0.782,0.784] | 0.111[0.116,0.120] |
| 0.877[0.875,0.879] | 0.173[0.177,0.184] | 0.751[0.750,0.753] | 0.103[0.106,0.109] |
| 0.897[0.895,0.899] | 0.16[0.168,0.175] | 0.774[0.773,0.776] | 0.100[0.103,0.106] |
| 0.875[0.872,0.877] | 0.149[0.158,0.166] | 0.758[0.757,0.760] | 0.095[0.101,0.104] |
| 0.878[0.877,0.880] | 0.131[0.146,0.152] | 0.766[0.764,0.767] | 0.095[0.099,0.102] |
| 0.9[0.899,0.902] | 0.146[0.156,0.163] | 0.775[0.775,0.778] | 0.096[0.101,0.104] |
| 0.877[0.875,0.88] | 0.127[0.134,0.14] | 0.754[0.752,0.755] | 0.096[0.099,0.103] |
| 0.875[0.874,0.879] | 0.17[0.18,0.188] | 0.742[0.739,0.743] | 0.096[0.099,0.103] |
| 0.864[0.862,0.867] | 0.157[0.168,0.176] | 0.740[0.740,0.743] | 0.099[0.103,0.107] |
| 0.877[0.875,0.88] | 0.211[0.22,0.228] | 0.738[0.735,0.738] | 0.086[0.089,0.092] |
| 0.857[0.853,0.858] | 0.178[0.183,0.193] | 0.732[0.730,0.734] | 0.087[0.090,0.094] |
| 0.852[0.849,0.854] | 0.191[0.198,0.209] | 0.750[0.748,0.752] | 0.074[0.081,0.084] |
| 0.869[0.866,0.871] | 0.213[0.215,0.225] | 0.736[0.734,0.737] | 0.082[0.085,0.088] |
| 0.851[0.846,0.852] | 0.24[0.245,0.256] | 0.737[0.737,0.740] | 0.094[0.097,0.101] |
